# Supplementary figures and images for: Charge neutralization as the major factor for the assembly of nucleocapsid-like particles from C-terminal truncated hepatitis C virus core protein
Source: PeerJ. 2016 Nov 9;4:e2670. doi: 10.7717/peerj.2670 (PMC5111903; doi:10.7717/peerj.2670)

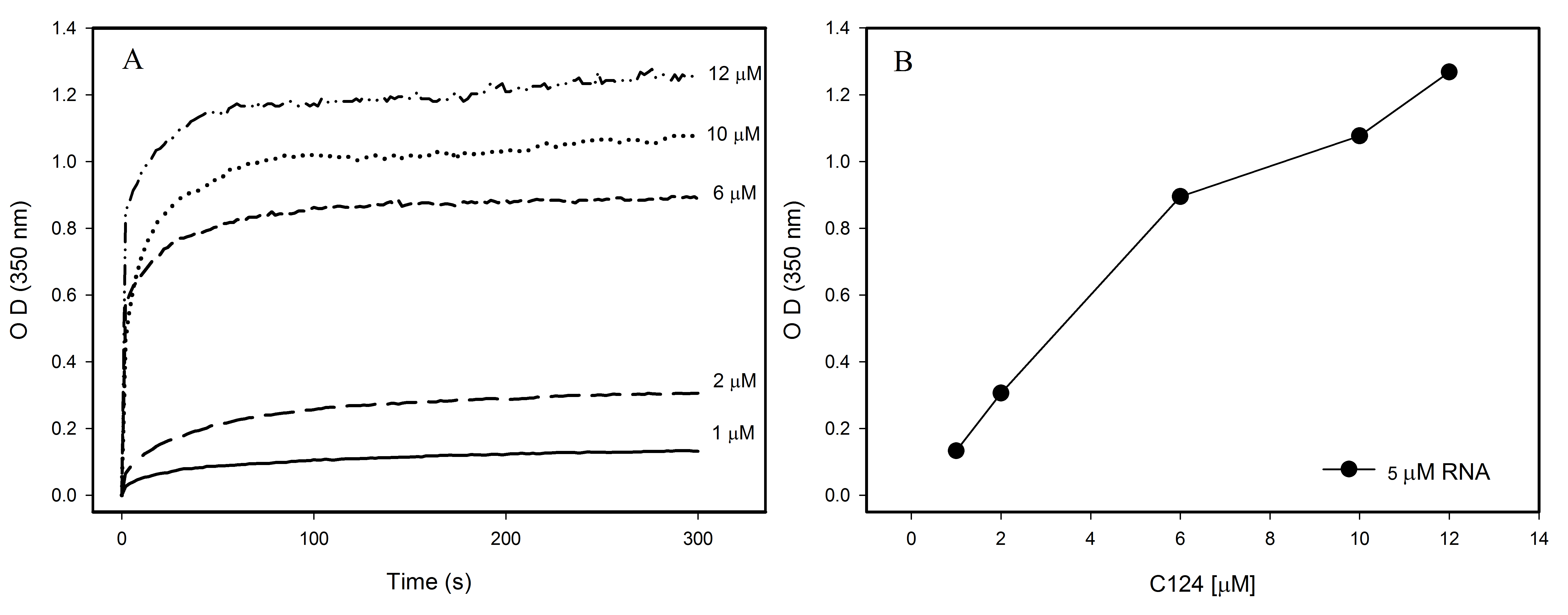

Supplement: Figure S1 — Interaction of HCV core protein with RNA and NLPs formation. (A) Temporal evolution studies of in vitro assembly of NLPs at different concentrations of C124 triggered by the addition of 5 µM RNA (SAF9343–59). (B) Plot of the maximum O.D. values at 350 nm as derived from the curves in A at different C124 concentrations. [file peerj-04-2670-s001.png]

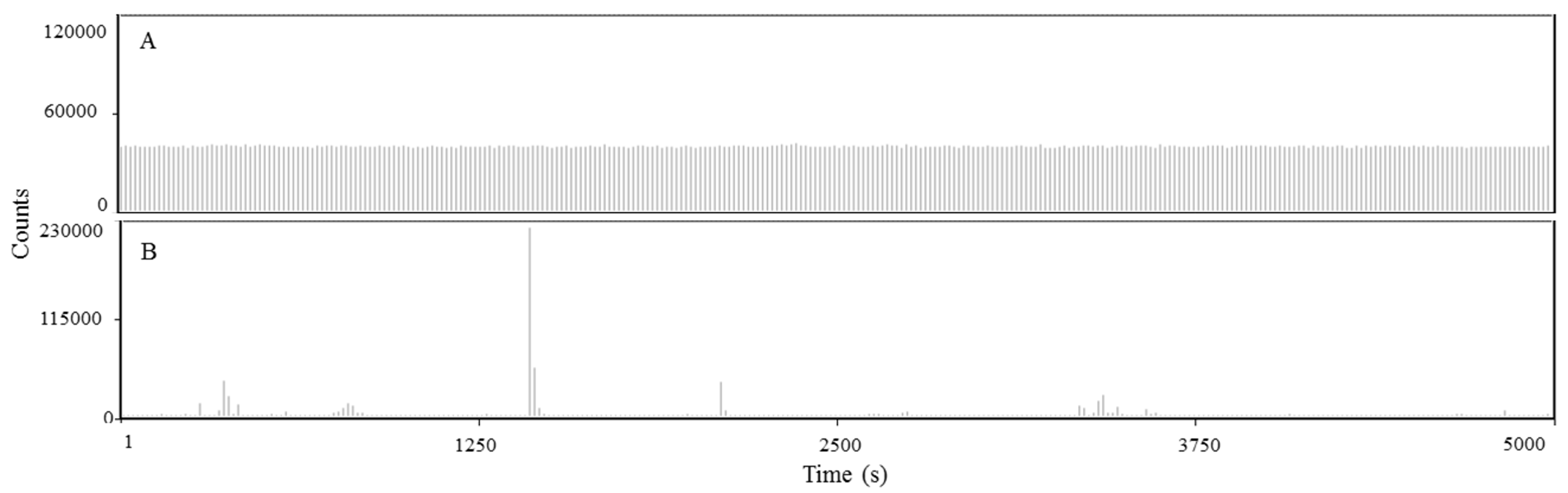

Supplement: Figure S2 — C-terminal truncated HCV core protein and unspecific nucleic acids (DNA) interaction analyzed by fluorescence correlation spectroscopy. Fluctuation of the fluorescence intensity of DNA labeled with Alexa-488 (100 nM) in the absence (A) or in the presence (B) of 1 µM of C124. The buffer used was 10 mM phosphate (pH 7.4) with 100 mM NaCl. [file peerj-04-2670-s002.png]

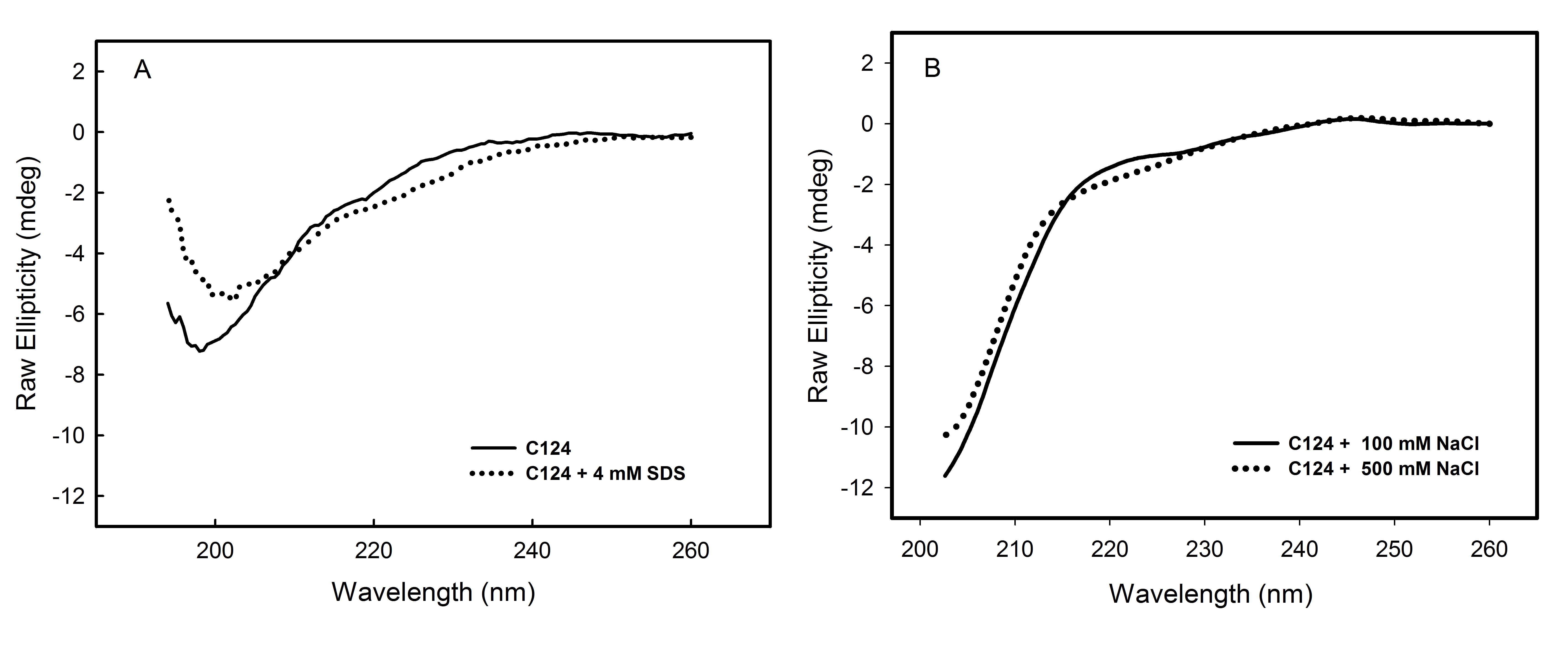

Supplement: Figure S3 — Analysis of the influence of SDS or NaCl on C124 structure. Circular dichroism spectra of C124 in the presence of 4 mM SDS (A) or 500 mM NaCl (B). The experiments were performed at room temperature. The protein concentrations were 3 µM (A) and 5 µM (B) and the measurements were performed utilizing 0.2 cm path length cells. [file peerj-04-2670-s003.png]
